# A Knowledge-Based Arrangement of Prototypical Neural Representation Prior to Experience Contributes to Selectivity in Upcoming Knowledge Acquisition

**DOI:** 10.3389/fnhum.2018.00111

**Published:** 2018-03-21

**Authors:** Hiroki Kurashige, Yuichi Yamashita, Takashi Hanakawa, Manabu Honda

**Affiliations:** ^1^Graduate School of Informatics and Engineering, The University of Electro-Communications, Tokyo, Japan; ^2^National Institute of Neuroscience, National Center of Neurology and Psychiatry, Tokyo, Japan; ^3^Integrative Brain Imaging Center, National Center of Neurology and Psychiatry, Tokyo, Japan

**Keywords:** fMRI, knowledge acquisition, sentence comprehension, learning, preplay, hippocampus, entorhinal cortex

## Abstract

Knowledge acquisition is a process in which one actively selects a piece of information from the environment and assimilates it with prior knowledge. However, little is known about the neural mechanism underlying selectivity in knowledge acquisition. Here we executed a 2-day human experiment to investigate the involvement of characteristic spontaneous activity resembling a so-called “preplay” in selectivity in sentence comprehension, an instance of knowledge acquisition. On day 1, we presented 10 sentences (prior sentences) that were difficult to understand on their own. On the following day, we first measured the resting-state functional magnetic resonance imaging (fMRI). Then, we administered a sentence comprehension task using 20 new sentences (posterior sentences). The posterior sentences were also difficult to understand on their own, but some could be associated with prior sentences to facilitate their understanding. Next, we measured the posterior sentence-induced fMRI to identify the neural representation. From the resting-state fMRI, we extracted the appearances of activity patterns similar to the neural representations for posterior sentences. Importantly, the resting-state fMRI was measured *before* giving the posterior sentences, and thus such appearances could be considered as preplay-like or prototypical neural representations. We compared the intensities of such appearances with the understanding of posterior sentences. This gave a positive correlation between these two variables, but only if posterior sentences were associated with prior sentences. Additional analysis showed the contribution of the entorhinal cortex, rather than the hippocampus, to the correlation. The present study suggests that prior knowledge-based arrangement of neural activity before an experience contributes to the active selection of information to be learned. Such arrangement prior to an experience resembles preplay activity observed in the rodent brain. In terms of knowledge acquisition, the present study leads to a new view of the brain (or more precisely of the brain’s knowledge) as an autopoietic system in which the brain (or knowledge) selects what it should learn by itself, arranges preplay-like activity as a position for the new information in advance, and actively reorganizes itself.

## Introduction

Activeness and spontaneity are characteristic features of the human brain. We act according to the psychological and physiological internal drives, regardless of whether we do so consciously or unconsciously, and we are not dominated by external stimuli. Even during knowledge acquisition, we do not indiscriminatingly receive information from the environment. We actively select such information according to internal factors.

Prior knowledge is one of the vital factors governing selectivity in knowledge acquisition. In some cases, prior knowledge facilitates the acquisition of additional information (Tse et al., 2007; van Kesteren et al., 2010a,b, 2013, 2014; Brod et al., 2016; Armelin et al., 2017). In other cases, prior knowledge inhibits the acquisition of information (Lipson, 1982; Alvermann et al., 1985; Kendeou and van den Broek, 2007). Sometimes, these contradicting effects cancel each other and there is no behavioral reflection; however, this competition can be recorded with brain imaging (Oren et al., 2017). One important notion is that knowledge acquisition is hampered when prior knowledge is sufficient to achieve a given goal (Sweegers et al., 2015), which implies that rationality is involved during knowledge acquisition.

Several studies have shown that, in order to update existing knowledge, one effectively explores the available information for the task and for the environment where the task will take place (Huang et al., 2008; Gureckis and Markant, 2012). This aspect of knowledge acquisition has been developed in machine learning and robotics studies as active learning (Krogh and Vedelsby, 1995; Tong and Koller, 2002; Settles, 2012). Curiosity is also an important epistemic value for our explorative behavior (Berlyne, 1966) and it determines whether or not the presented information is assimilated (Kang et al., 2009; Gruber et al., 2014). Interestingly, humans seem to have the greatest curiosity for an information that gives them an intermediate feeling of knowing (Kang et al., 2009), rather than a complete unknown. This suggests that the recognition of a deficit in knowledge encourages selective acquisition of this knowledge for efficient completion.

Anticipation of the use of information also facilitates knowledge acquisition through sleep consolidation (Fischer and Born, 2009; Wilhelm et al., 2011; van Dongen et al., 2012). Additionally, the application of knowledge strengthens knowledge acquisition (Karpicke and Roediger, 2008; Karpicke and Blunt, 2011). This is relevant because information that is used should have a greater value than information that is not used.

The evidence mentioned above implies that, based on prior knowledge, we actively select the information that is missing and/or would improve our knowledge, and thus, should be learned. In other words, our brain seeks novel information to complement, augment, and upgrade current knowledge. However, how the brain mechanistically and computationally realizes such selective acquisitions of knowledge, and how that knowledge is upgraded, remains unknown.

Here, we hypothesize that prior knowledge guides the selectivity of knowledge acquisition by arranging prototypical neural representations prior to concrete experience. The observations of “preplay” activity in the rodent hippocampus (Dragoi and Tonegawa, 2011, 2013) support this hypothesis. Preplay is a distinguishing spontaneous neural activity observed during periods of rest that organizes before an experience and becomes an actual representation after the experience.

In a conventional view, neural representation forms during and after learning or an experience. Following a learning experience, several studies analyzing the rodent hippocampus (Louie and Wilson, 2001; Lee and Wilson, 2002; Foster and Wilson, 2006; Diba and Buzsáki, 2007; Carr et al., 2011; Wu et al., 2017) and the rodent entorhinal cortex (Ólafsdóttir et al., 2016; O’Neill et al., 2017) have reported the appearance of learned neural representations during spontaneous activity, namely replay. Using functional magnetic resonance imaging (fMRI), several studies have suggested the existence of replay-like activity in the human brain (Deuker et al., 2013; Staresina et al., 2013; Tambini and Davachi, 2013; Schlichting and Preston, 2014; de Voogd et al., 2016; Hermans et al., 2017). These studies identified positive relationships between the strength of replay-like activity in offline processing and performance during memory recall.

As opposed to replay activity, preplay activity suggests that the neural representation forms *before* the experience, although the relevance of this activity has been controversial (Silva et al., 2015; Grosmark and Buzsaki, 2016). Therefore, we naturally consider the predictive or proactive characteristics in preplay activity. In the present study, we investigated the effects of preformed neural representations on knowledge acquisition. Our main hypothesis was that prototypical neural representations that are arranged prior to experience facilitate knowledge acquisition. Moreover, we hypothesized that it depends on existence of prior knowledge. To these ends, we executed a 2-day sentence comprehension experiment in which the subjects learned prior and posterior sentences on days 1 and 2, respectively. These sentences were difficult to understand on their own, but the association between prior and posterior sentences was meant to facilitate understanding. We measured the resting-state fMRI (rsfMRI) prior to the learning of the posterior sentences. Then, after learning the posterior sentences, we measured the induced fMRI during the presentation of posterior sentences to identify the neural representations of them. Based on correlation between this neural representation and activity pattern in the rsfMRI, we defined the prototypical neural representation. We compared the appearance strengths of the prototypical neural representation in the hippocampal and entorhinal rsfMRI with the ratings of sentence comprehension. This gave a positive correlation between these two variables in the condition where the prior sentences were available for understanding. Moreover, since the previous rodent study suggested that preplay activity is stabilized and established through experience (Grosmark and Buzsaki, 2016), we examined this stabilization effect. Moreover, we showed that the entorhinal cortex, rather than the hippocampus, contributes to the observed effect. In addition, we conducted a whole-brain analysis to explore brain regions involved with the effect beyond the hippocampus and entorhinal cortex. Finally, we proposed a possible mechanism to explain our findings.

## Materials and Methods

### Subjects

Seventeen right-handed subjects (7 females; mean age 21.4 years; age range 20–23 years) without a history of a neurological or psychiatric disease participated in this study. All subjects were native Japanese speakers and had normal or corrected-to-normal vision. This study was carried out in accordance with the recommendations of the institutional ethics committee of the National Center of Neurology and Psychiatry (NCNP) with written informed consent from all subjects. All subjects gave written informed consent in accordance with the Declaration of Helsinki. The protocol was approved by the institutional ethics committee of the National Center of Neurology and Psychiatry (NCNP). One male subject was excluded from the final analysis because he fell asleep during the experiment on day 1.

### Experimental Procedures

**FIGURE 1:**
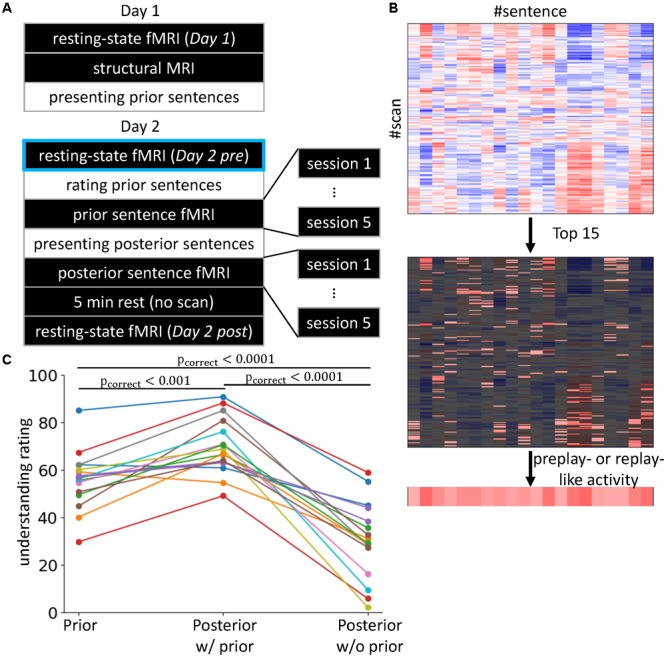
Experimental setting and behavioral results. **(A)** 2-day Experiment. The black rectangles indicate the in-scanner tasks, while the tasks described in the white rectangles were executed outside of the scanner. The *Day 2 pre* rsfMRI enclosed with light blue is the main focus of the present study, in which we investigate preplay-like activity. **(B)** Definitions of preplay- and replay-like activity. We calculated the Fisher-transformed correlations between the rsfMRI and neural representations of sentences (upper). Then, we selected the highest 15 values (middle) and averaged them for each sentence (lower). The resultant value was defined as the intensity of preplay- or replay-like activity for each sentence. **(C)** Difficulties in understanding the sentences. We compared the understanding ratings by subjects for the prior sentences (left), the posterior sentences associated with prior sentences (center), and the posterior sentences without any association (right). Each line corresponds to a subject. The colors are only for visualization purposes.

The experiment consisted of two continuous days (**Figure 1A**). The subjects were tasked with understanding 10 prior and 20 posterior sentences on days 1 and 2, respectively. Individually, each sentence was difficult to understand. However, several posterior sentences were associable with prior sentences, and therefore they became more understandable through the associations.

On day 1, we first measured the rsfMRI (*Day 1*) and then acquired a T1-weighted structure MRI. Next, the subjects were removed from the MRI scanner and engaged in the sentence comprehension task for prior sentences. On day 2, we first acquired the rsfMRI (*Day 2 pre*). Then, outside the scanner, we presented the same prior sentences as on day 1 and asked the subjects to perform the rating task. Next, the subjects re-entered the scanner and, while the fMRI measurements were being performed, subjects were asked to imagine the meaning of the prior sentences (see section “Stimulus-Induced fMRI”). The subjects were removed from the scanner and given the sentence comprehension task for posterior sentences. This was followed by a question and answer session regarding the inferred association between prior and posterior sentences. Then, the subjects re-entered the MRI scanner and were asked to imagine the meaning of posterior sentences while the fMRI measurements were taking place. After a 5-min rest with no scans, we acquired another rsfMRI (*Day 2 post*).

### Prior and Posterior Sentences

We extracted 20 propositional sentences from Japanese-translated literature of various disciplines and adjusted them to be used as prior sentences. In Supplementary Table S1, we show the English versions of the sentences in published translated materials (if they exist) or those translated by us with bibliographic information. Then, we created 20 posterior sentences beginning with “Therefore” or “This is because” where the thematic words were replaced with pronominal expressions. Originally, we intended to have an association between each posterior sentence and each prior sentence. However, instead we considered which prior and posterior sentences the subjects thought were associated (see section “Posterior Sentence Presentation”), as we did not focus on the “ground truth” of correspondence. Both prior and posterior sentences were difficult to understand on their own, but identifying associations between the sentences facilitated their understanding.

### Prior Sentence Presentation

We applied the sentence comprehension task for the 10 prior sentences that were randomly selected from the 20 prior sentences for each subject. For each trial, we presented one prior sentence on the display and asked the subjects to deeply consider the meaning of the sentence. When the subjects felt they had enough time for consideration, they pressed a key to proceed to the next sentence. After finishing their deep analysis of all 10 sentences, the subjects proceeded to the next session, which was the same task but with the sentences in a shuffled order. The subjects repeated the sessions for up to 60 min. The task was constructed using Win32 API programming.

### Prior Sentence Rating

The subjects rated the prior sentences presented on day 1 for four items: (1) the understanding of prior sentences, (2) the expected increment of prior understanding, (3) the importance, and (4) the subjective need. For (1), subjects assessed the depth of their understanding of prior sentences from 0 to 100. These self-ratings were used as the indexes of understanding of prior sentences for subsequent analyses. For (2), subjects reported their expectations of change in the depth of understanding of prior sentences from 0 to 100, assuming that they would be given the new related sentences. For (3), subjects answered the subjective importance of knowing the new sentences relating to prior sentences on a five-point scale. For (4), subjects answered how much they wanted to know the new sentences that were related to prior sentences on a five-point scale. The task was completed with a wxWidgets GUI application constructed by the wxPython module^1^ in Python. The responses were acquired using sliders for (1) and (2), and radio buttons for (3) and (4).

### Posterior Sentence Presentation

The sentence comprehension task was applied for 20 posterior sentences. This task was executed using a wxWidgets GUI application made with the wxPython module in Python. For this task, we presented all 20 sentences and the corresponding sliders on one screen. Subjects were required to understand them and to rate their degree of understanding of the posterior sentences from 0 to 100. As with prior sentences, we used those self-ratings as the indexes of understanding of the posterior sentences in subsequent analyses. Before the task, the subjects were told that 10 of the 20 sentences were to be posterior to the prior sentences and that the prior sentences would be useful to understand the posterior sentences. Subjects were also instructed to memorize the sentences, as they would only be briefly presented as the stimuli in a subsequent fMRI scanning.

After the comprehension and rating tasks, the subjects were asked which prior sentences they thought were associated with posterior sentences. For each posterior sentence, the subjects selected one prior sentence or N/A from a pull-down menu. If a subject thought that several posterior sentences were associated with the same prior sentence, they were required to give overlapping answers. As a result, the number of N/A answers for each subject did not necessarily add up to 10. As discussed in Section “Prior and Posterior Sentences,” we considered the associations described by the subjects as the true associations for the following analyses.

### MRI Scanning

We used a 3T MRI scanner (Trio, Siemens Medical Solutions, Erlangen, Germany) with an 8-channel head coil for all measurements. Structural images were acquired with a T1 weighted 3D magnetization-prepared-rapid-gradient-echo (MPRAGE) sequence (flip angle = 8°; voxel size = 1 mm isotropic; TR = 2000 ms; TI = 990 ms; TE = 4.38 ms; number of voxels = 208 × 256 × 208). fMRI images were acquired with a T2^∗^-weighted echo planar imaging sequence (flip angle = 90°; voxel size = 3 mm isotropic with no slice gap; TR = 3000 ms; TE = 30 ms; number of voxels = 64 × 64 × 44). The slices were acquired in interleaved order.

### Resting-State fMRI

For each session of rsfMRI, 200 volumes of images were acquired. Since TR = 3 s, the total acquisition time for each session was about 10 min. During imaging, a fixation point centered on a gray background was presented on the screen using the projector (DLA-HD10K, JVC, Kanagawa, Japan). The subjects saw the screen through the mirror equipped with a head coil of the scanner. We instructed the subjects to gaze onto the fixation point and to think of nothing in particular.

### Stimulus-Induced fMRI

To identify the neural representations of the prior and posterior sentences, we measured the fMRI of the subjects while they judged whether the sentences were semantically related to two-character Japanese words. Both sets of fMRI measurements for prior and posterior sentences consisted of five sessions. A session for prior sentences contained 10 trials, each of which corresponded to one prior sentence and included a rest block (two scans), a sentence presentation block (six scans), and a word presentation and response block (one scan). We added three rest scans at the beginning and end of the session. Therefore, one session for prior sentences consisted of 96 total scans. The session for posterior sentences was similar, but the number of trials was 20, because there were 20 posterior sentences. Thus, one session for posterior sentences consisted of 186 total scans. The setting of the projector, screen, and mirror was same as for the measurement of rsfMRI. To acquire a response in the response block, we used the MRI compatible button box with four buttons for each hand (HHSC-2x4-C, Current Designs, Philadelphia, PA, United States).

In the sentence presentation block, one sentence was displayed and the subjects were asked to imagine the meaning of the sentence. They were also asked to be ready for the successive word presentation and response block. Additionally, subjects were instructed to return their gaze to the fixation point after grasping the sentence as rapidly as possible.

In the word presentation and response block, a word was presented during the first 2.5 s of the block, and subjects were asked to push the button within this period. Subjects were instructed to press the button with their right index finger if they thought that the word was semantically related to the sentence that was presented immediately before. If the subjects did not identify a relationship, they were asked to press the button with their right middle finger. In the remaining 0.5 s, the circle symbol was used as a feedback indicator that the button was pressed with the index finger, and the cross symbol was used as a feedback indicator that the button was pressed with the middle finger. If the subject did not press with any finger, a hyphen was used as a feedback indicator. The words were randomly chosen from the 3,000 most frequent two-character Japanese words recorded in the balanced corpus of contemporary written Japanese (Maekawa et al., 2014). The purpose of this task was to task the subjects with keeping the images of the meanings of presented sentences during the sentence presentation block. Since the subjects had to respond to the words within a short duration, this task forced them to keep the images to ready to judge semantic relatedness with the words. The resultant responses generated by the subjects were not relevant to this study. Therefore, we did not analyze the responses.

### Preprocessing of MRI Data

Preprocessing was completed primarily using SPM12 (Wellcome Trust Centre for Neuroimaging, England, United Kingdom) MATLAB toolbox (Mathworks, Natick, MA, United States) and custom-made Python scripts. To make the individual cortical and subcortical atlases, we also used Freesurfer (Version 5.3.0^2^) and FSL (FMRIB Software Library Version 5.0.6^3^).

The same pipeline of preprocessing was applied for both of stimulus-induced and resting-state fMRI. All steps were executed by running functions in SPM12 from custom-made MATLAB scripts, and not from GUI. The three initial scans for each series of functional imaging data were discarded to exclude the T1-saturated images. As the first step of preprocessing, the slice time correction was applied. Then, the realign and reslice procedure was performed using the mean slices of all sessions as a reference, including resting-state and stimulus-induced imaging. Next, co-registration of the structural images with the mean functional images was completed. After that, we divided preprocessing pipeline into two: one for main region of interest (ROI) analysis and the other for additional exploratory whole-brain analysis.

For the main ROI analysis, the functional images were spatially smoothed with FWHM = 5 mm. The use of spatial smoothing in multi-voxel pattern analysis (MVPA) is controversial because it is useful for noise reduction but it also reduces the dimensionality of the data. However, several recent studies support to the use of smoothing (Op de Beeck, 2010; Gardumi et al., 2016; Hendriks et al., 2017), and the effective dimensionality was large enough in our analyses even if we applied the smoothing. Therefore, we chose to use spatial smoothing. We did not normalize to a standard brain image since we analyzed the data in individual atlases to avoid distortion resulting from the normalization.

To identify the individual brain atlases, the recon-all procedure in Freesurfer was applied to obtain two kinds of cortical atlases (Desikan et al., 2006; Destrieux et al., 2010) and one subcortical segmentation (Fischl et al., 2002) for each subject. In the present study, the Desikan–Killiany atlas was used as the cortical atlas. To register the individual atlas with the functional images, atlas mgz files were first transformed to nifti files using the mri_convert command in Freesurfer. Then, atlas nifti files were resliced using the reslice_nii function in Tools for NIfTI and ANALYZE image^4^. Then, transformation matrix was identified from resliced orig image in Freesurfer to the structural image that was already co-registered to the mean functional image using the flirt command in FSL. Finally, individual atlas was constructed in the functional images by corresponding the atlas images to the functional images through the transformation matrix described above.

For the exploratory whole-brain analysis, we executed the segmentation and normalization to map the images into the Montreal Neurological Institute (MNI) standard space. Then, the functional images were weakly smoothed (FWHM = 3 mm).

### Representational Similarity Analysi*s*

To measure the intensities of preplay-like and replay-like activities in the rsfMRI, we adopted the representational similarity analysis (Kriegeskorte, 2008; Kriegeskorte and Kievit, 2013) between the stimulus-induced and resting-state fMRI data. The aim of this analysis was to detect spontaneous activation of the patterns similar to neural representations for sentences in rsfMRI.

Previous studies showed that an experience could induce changes in rsfMRI pattern, which is found not only in areal level but also in voxel level (Albert et al., 2009; Lewis et al., 2009; Taubert et al., 2011; Guidotti et al., 2015). Especially, the MVPA approach showed that task learning increases the similarity between voxel patterns in rsfMRI and in task fMRI (Guidotti et al., 2015). An important point is that this was shown through applying MVPA classifier trained with task fMRI into rsfMRI, which is similar to what we did in the present study. In line with basically same idea, several studies used the representational similarity analysis to compare the rsfMRI or task-irrelevant fMRI with task fMRI (Staresina et al., 2013; de Voogd et al., 2016). Those studies also reported that the similarity between voxel patterns in rsfMRI and in task fMRI was related to the task performance or task condition.

In addition, a recent study using MVPA suggested that brain activity patterns that are induced by sentences are predictable from the patterns that are induced by the word that compose the sentences (Anderson et al., 2017). Moreover, it has been suggested that the representational similarity analysis can successfully quantify the semantic difference and similarity of word stimuli (Bruffaerts et al., 2013; Carota et al., 2017). Taken together, this analysis is applicable to the aim of this study.

**FIGURE 2:**
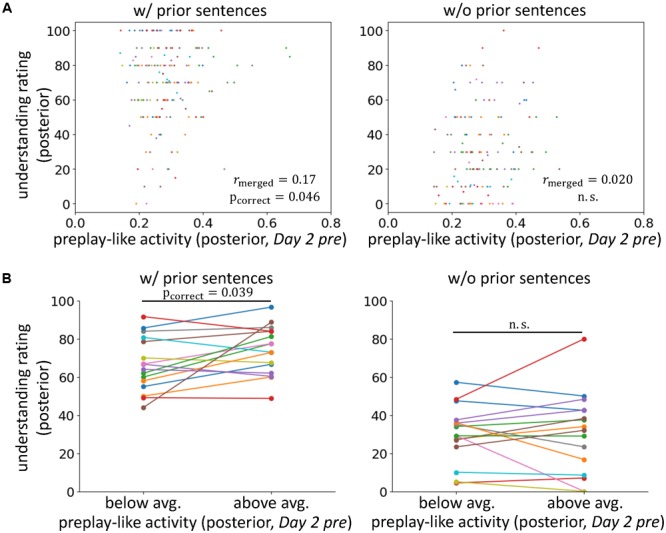
Preplay-like activity facilitates the understanding of posterior sentences when prior knowledge is available. **(A)** Correlations between the intensity of preplay-like activity (in *Day 2 pre* rsfMRI) for posterior sentences and the understanding rating for posterior sentences. The posterior sentences were associated with prior sentences (left; w/prior) or not (right; w/o prior). Each color of dots corresponds to a subject. The notation of the *y*-axis title “understanding rating (posterior)” means “value of the understanding rating for posterior sentences.” The notation of the x-axis title “preplay-like activity (posterior, *Day 2 pre*)” means “intensity of preplay-like activity in *Day 2 pre* rsfMRI for posterior sentences.” We used similar notations throughout all figures in this paper. **(B)** Comparison of the understanding ratings of posterior sentences possessing above-average and below-average preplay-like activity (in *Day 2 pre* rsfMRI). The posterior sentences were associated with prior sentences (left; w/prior) or not (right; w/o prior).

**FIGURE 3:**
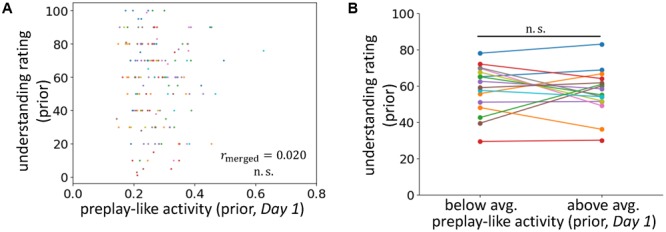
No facilitating effect for the understanding of prior sentences. **(A)** Correlations between the intensity of preplay-like activity (in *Day 1* rsfMRI) for prior sentences and the understanding rating for prior sentences. **(B)** Comparison of the understanding ratings of prior sentences possessing above-average and below-average preplay-like activity (in *Day 1* rsfMRI).

**FIGURE 4:**
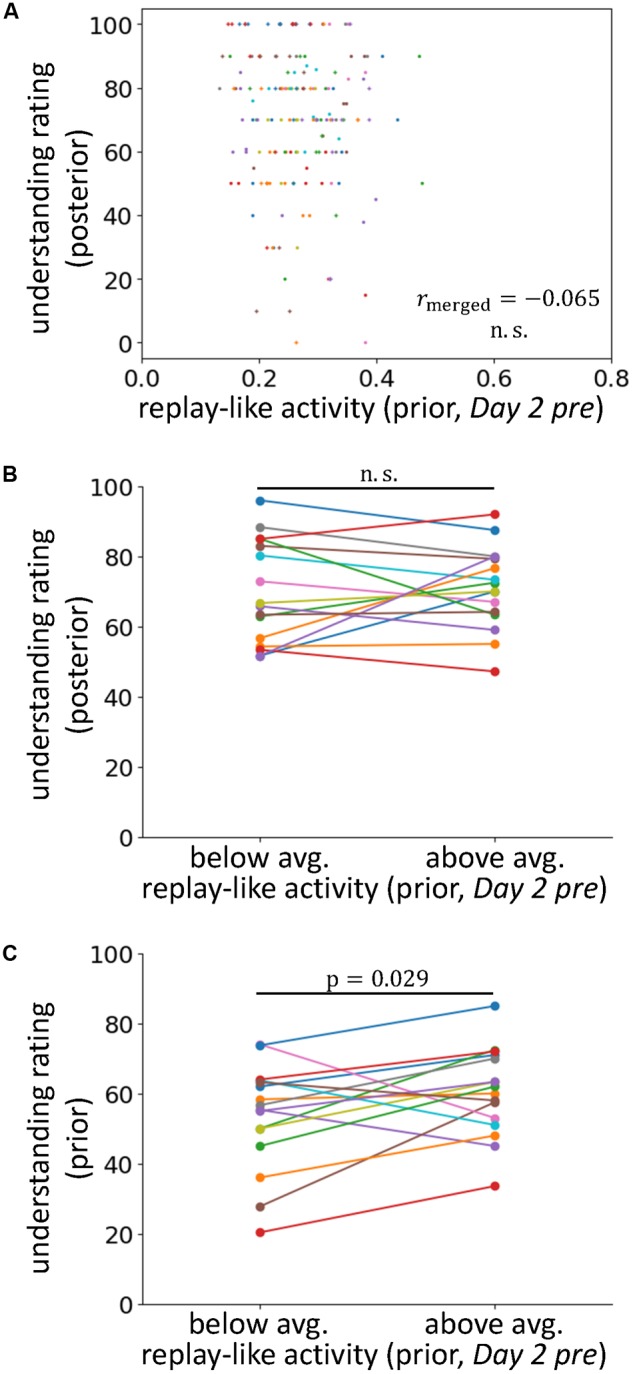
The effects of replay-like activity for prior sentences. **(A)** Correlations between the intensity of replay-like activity (in *Day 2 pre* rsfMRI) for prior sentences and the understanding rating for posterior sentences. **(B)** Comparison of the understanding ratings of posterior sentences whose associating prior sentences possessed above-average and below-average replay-like activity (in *Day 2 pre* rsfMRI). **(C)** Comparison of the understanding ratings of the prior sentences possessing above-average and below-average replay-like activity (in *Day 2 pre* rsfMRI).

**FIGURE 5:**
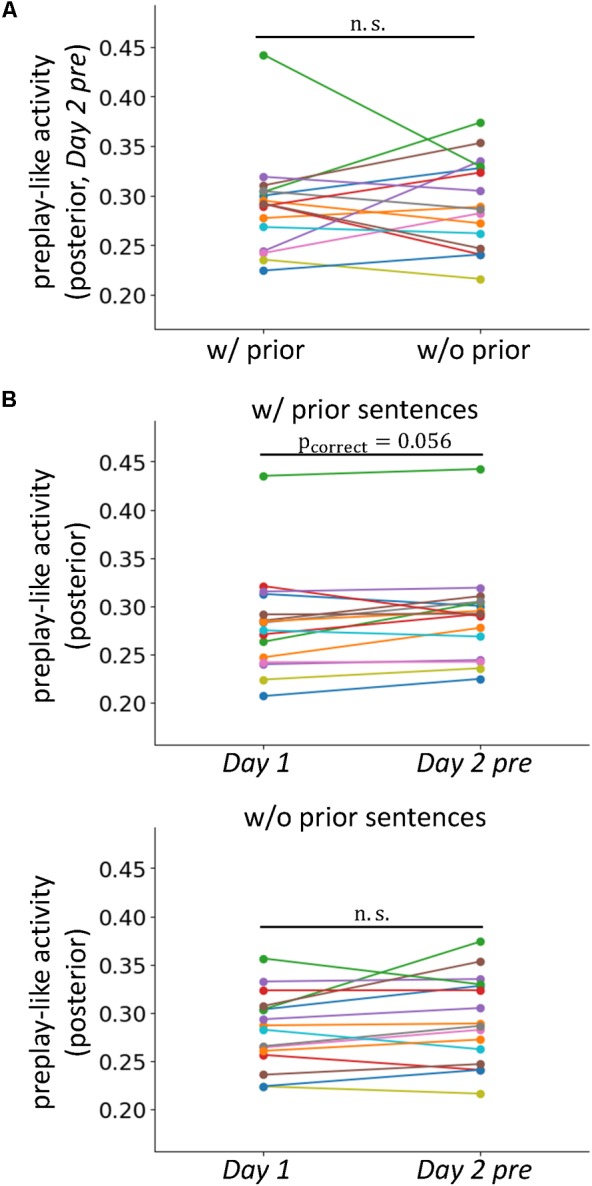
Prior knowledge does not result in a newly organized pattern of neural activity. **(A)** Comparison of the intensities of preplay-like activity (in *Day 2 pre* rsfMRI) for posterior sentences with and without associated prior sentences. **(B)** Comparisons of the intensities of preplay-like activities for posterior sentences in *Day 1* rsfMRI and *Day 2 pre* rsfMRI. The upper panel shows the case in which the associated prior sentences existed. The lower panel shows the case in which the associated prior sentences did not exist.

**FIGURE 6:**
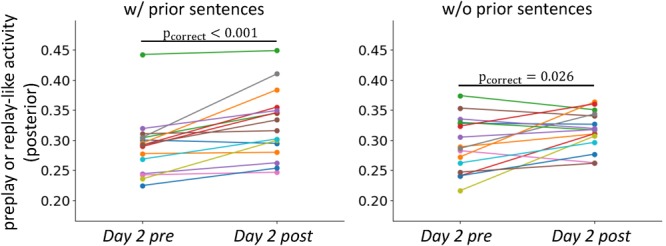
Stabilization of neural representations after the experience. Comparisons of the intensities of preplay- and replay-like activities for posterior sentences in *Day 2 pre* rsfMRI and *Day 2 post* rsfMRI, respectively. The left panel shows the case in which the associated prior sentences existed. The right panel shows the case in which the associated prior sentences did not exist.

**FIGURE 7:**
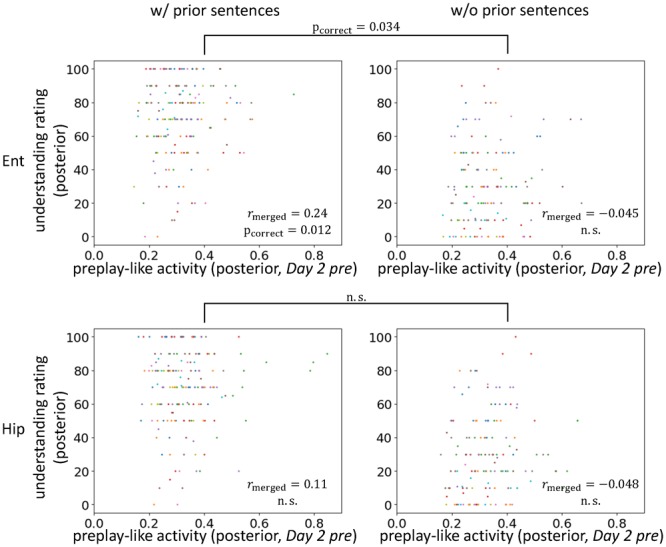
Entorhinal cortical contribution of facilitating understanding by preplay-like activity. Correlation between the intensity of preplay-like activity (in *Day 2 pre* rsfMRI) for posterior sentences and the understanding rating for posterior sentences. The posterior sentences were associated with prior sentences (**left**; w/prior) or not (**right**; w/o prior). The entorhinal cortex (**upper**; Ent) and the hippocampus (**lower**; Hip) are compared. Each color of dots corresponds to a subject.

Using SPM12, we applied first level analysis for the prior and posterior sentence fMRIs to estimate the beta maps for each stimulus. Using the spm_getSPM function in SPM with the contrast including the beta maps belonging to the same sentence, we obtained a t-statistics map for each sentence. For the main ROI analysis, we regarded each t-statistics map masked by the anatomical ROI as the neural representation for each sentence. The ROIs were defined with the individual atlas mentioned above. Since the previous rodent studies reported replay activity not only in the hippocampus but also in the entorhinal cortex (Ólafsdóttir et al., 2016; O’Neill et al., 2017), we used the merged ROI of the hippocampus and entorhinal cortex (**Figures 2–6**) and the separated hippocampal and entorhinal ROIs (**Figure 7**). As we had 10 prior and 20 posterior sentences, we obtained 10 and 20 representations for prior and posterior sentences, respectively. To exclude the non-informative voxels, we discarded the voxels with the lowest 3% variances of the *t*-values across prior (or posterior) sentences.

For the rsfMRI, we first standardized the time course for each voxel to the *z*-score to remove spatial biases. Next, we measured the correlation between the representation defined above and the voxel pattern for each time slice in the rsfMRI. Since there were 10 and 20 representations for prior and posterior sentences, respectively, this resulted in 10 and 20 time courses of correlations, each of which corresponded to a sentence.

In the present study, we defined the mean of the top *N*_top_ Fisher *Z*-values of the correlations in the scans of rsfMRI as the intensity of preplay-like or replay-like activity for the representation (**Figure 1B**). The aim of this average operation was noise reduction. Therefore, the value of *N*_top_ had to be large enough to resist a noise. At the other extreme, baseline activity would hide the preplay-like activity and make it invisible if we used overall mean. Therefore, we needed to choose an adequate value of *N*_top_. We theoretically decided the best value of *N*_top_ based on the estimation of variance of preplay-like activity over the posterior sentences. The variance of the intensities of preplay-like activity is the sum of the noise-derived component and the signal component. Since the noise-derived component is proportional to $N_{\mathrm{top}}^{–1}$, we identified the signal component as the residual of linear fitting of the variance of the preplay-like activity to $N_{\mathrm{top}}^{–1}$. Then, we assigned the value of *N*_top_ as the point for maximum signal component. This resulted in 14.81 ± 3.52 (mean ± SD; *n* = 16). Thus, we set *N*_top_ = 15. Note that we never used any information about the understanding of the sentences in this process. Additionally, we found that a wide range of *N*_top_ values ([10,15,...,80]) gave qualitatively invariant main results (shown in **Figure 2A**) and the “15” was located near the edge of such a range. Therefore, we also used the median of the range (i.e., *N*_top_ = 45) and have showed this result in the Supplementary Material. Note that this theoretical estimation explained above might not be exact since we premised the i.i.d. condition. However, it is good approximation when exact statistical model is not available.

In the present study, we mainly used the results of the representational similarity analysis for comparison with the understanding rating of the prior and posterior sentences. Therefore, we treated data combinations of rsfMRI sessions (*Day 1, Day 2 pre*, and *Day 2 post*), stimulus-induced fMRI sessions (prior and posterior sentences), and understanding ratings (prior and posterior sentences). We included a table to show which combination we used to generate each figure (Supplementary Table S2).

In addition to the main ROI analysis, we also conducted whole-brain analysis. To define the intensity of preplay-like activity for each voxel, again we applied the representational similarity analysis that was same to the analysis described in this section but to small cube neighboring each voxel aligned in the MNI space. See the next section for the detailed explanation of the whole-brain analysis.

### Exploratory Analysis of the Whole Brain

To investigate the preplay-like activity across the whole brain, we adopted the method similar to the searchlight approach (Kriegeskorte et al., 2006). We executed this analysis only for the pair of the preplay-like activity for the posterior sentences in *Day 2 pre* rsfMRI and the understanding of the posterior sentences. To obtain t-statistics maps for the posterior sentences, the processing described in Section “Representational Similarity Analysis” was used but in MNI standard space. We considered a pattern of each t-statistics map within the 5 × 5 × 5 cube of voxels neighboring a voxel as the neural representation of corresponding posterior sentence at the voxel. As with the ROI analysis mentioned above, we measured the correlation between the neural representation and the voxel pattern for each time slice in the rsfMRI within each cube. Then, for each neural representation, we defined the mean of the top *N*_top_ Fisher *Z*-values of the correlations as the intensity of preplay-like activity for each voxel. We determined that *N*_top_ = 13 since the value resulted from the estimation of variance was 13.13 ± 2.52 (mean ± SD, *n* = 16). This resulted in the whole-brain map of the preplay-like activity for each posterior sentence.

To compare those maps with the understanding of the posterior sentences, the general linear model (GLM) analysis was completed using the randomize command in FSL, in which we set the understanding ratings and the categorical variables for the subjects as the explanatory variables. The number of permutations was 5,000. We used the mask of the gray matter and subcortical nuclei that was constructed from the Freesurfer atlas. To identify significant cluster, we utilized the threshold-free cluster enhancement method with default parameter values (Smith and Nichols, 2009).

### Statistical Analyses

**FIGURE 8:**
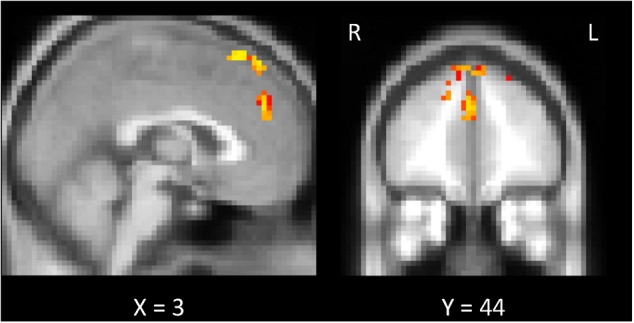
Exploratory analysis of the whole brain. Comparison of the “w/prior” and “w/o prior” posterior sentences. In both cases, the correlations between the preplay-like activity (in *Day 2 pre* rsfMRI) for the posterior sentences and the understanding rating for the posterior sentences were calculated for each voxel. Then, these correlations were compared. The significant voxels (*p* < 0.05) are shown under the FWE- and Bonferroni-corrections for the number of voxels and the three conditions (r_w/prior_ > 0, r_w/o prior_ > 0, r_w/prior_ > r_w/o prior_), respectively.

For statistical tests, we mainly used SciPy^5^ and statsmodels^6^ modules in Python. We executed the paired *t*-tests using the ttest_rel function in SciPy. We completed the analysis of variance (ANOVA) and the analysis of covariance (ANCOVA) using the ols function in statsmodels where repeated designs were modeled using the subject as a categorical variable. We utilized the multipletests function in statsmodels with Bonferroni methods for correcting for multiple comparisons. We also used the aov function and the multcomp package in R^7^ for executing ANOVA and Tukey *post hoc* tests. When we analyzed our main hypothesis (the prototypical neural representations arranged prior to experience facilitate knowledge acquisition), we utilized one-sided tests (**Figures 2, 4C, 7, 8**). In addition, we used one-sided tests if we drew a conclusion based on the acceptance of the null hypothesis because it was more conservative (**Figures 3, 4AB, 5**), and if a prediction from a previous study was available (**Figure 6**). In all other cases, two-sided tests were used.

To test our main hypothesis, we first calculated the correlation between the intensities of preplay-like activity and the degrees of sentence comprehension (the understanding ratings) for each subject. Then, we merged them across subjects. The number of posterior sentences associated with prior sentences was different across subjects, as discussed in the section “Posterior Sentence Presentation.” This meant that the sample sizes for calculating the correlations varied and, therefore, we needed to weight the statistical values based on the sample sizes. We obtained the weighted statistics and the distribution governing them using the following method borrowed from the meta-analytic field (Mosteller and Bush, 1954; Rosenthal, 1978, 1991; Hedges and Olkin, 1985). Here we defined the correlation for *i*th subject as *r*_i_. First, we calculated the Fisher transformation of each correlation coefficient: Z_i_ = 0.5ln(1+r_i_/1–r_i_). When the sample size is *n*_i_, the weighted sum is ζ = $\underset{i}{\Sigma }$w_i_Z_i_, where w_i_ = (n_j_–3)/$\underset{j}{\Sigma }$(n_j_–3). Thus, the merged correlation becomes r_merged_ = (e^2ζ^–1)/(e^2ζ^+1). It is known that the standard error of *Z*_i_ is 1/$\sqrt{n_{i}-3}$ (Snedecor and Cochran, 1989). Therefore, the standard error of ζ becomes SE_ζ_ = 1/$\sqrt{\underset{i}{\Sigma }(n_{i}-3)}$, considering the results of error analyses (Taylor, 1997). We can test the null hypothesis as ζ = 0 (i.e., r_merged_ = 0) by calculating the deviation of ζ/SE_ζ_ from standard normal distribution. For comparison between ζ_1_ and ζ_2_, we utilized the fact that (ζ_1_–ζ_2_)/$\sqrt{SE_{\zeta_{1}}^{2}+SE_{\zeta_{2}}^{2}}$ has the standard normal distribution under the null hypothesis (i.e., ζ_1_ = ζ_2_).

## Results

In the present study, we investigated the effects of prior knowledge on the selectivity of knowledge acquisition through the spontaneous arrangement of neural activity prior to an experience. To this end, we applied a 2-day sentence comprehension task. In this task, 10 prior sentences that were presented on day 1 were considered to aid in the understanding of some of 20 posterior sentences presented on day 2.

### Behavioral Results

Originally, there were pre-established associations between prior and posterior sentences. However, the possibility remained that subjects would fail to grasp these associations. Therefore, we compared our associations with those made by the subjects during the presentation of posterior sentences. If the subjects thought that no prior sentence corresponded to the posterior sentence, they chose the “N/A” option from the pull-down menu of the 10 prior sentences presented on day 1. The proportion of concordance to our assumptions was not 1 but 0.70 ± 0.12 (mean ± SD, *n* = 16). Additionally, the number of answers that were not N/A was not 10, but 11.1 ± 1.5 (mean ± SD, *n* = 16). As discussed in Section “Prior and Posterior Sentences,” we considered the associations made by each subject to be true.

As mentioned previously, one premise of this study was that the associations between prior and posterior sentences facilitate understanding. This means that prior sentences alone (before association with posterior sentences) and the posterior sentences alone (without any association to prior sentences) were more difficult to understand compared with the posterior sentences that were associated with prior sentences. To confirm that this premise was satisfied, we compared the degrees of subjective ratings of understanding for three conditions of sentence presentations (“Prior,” “Posterior w/prior,” and “Posterior w/o prior”; **Figure 1C**). We observed the main effect of the conditions of sentence presentation using one-way repeated measures of ANOVA, for which the dependent variables were the means of the reported degrees of understanding averaged within each condition [F(2, 30) = 69.78, p < 10^-11^]. Tukey’s *post hoc* test showed “Posterior w/prior” > “Posterior w/o prior” (t = 11.67, p < 0.0001) and “Posterior w/prior” > “Prior” (t = 4.22, p < 0.001). This result indicates that prior sentences did facilitate the understanding of posterior sentences when an association was present. Additionally, we observed “Prior” > “Posterior w/o prior” (t = -7.45, p < 0.0001). This result indicates that the posterior sentences themselves were more difficult to understand compared with the prior sentences. We also executed the paired *t*-test corrected by the Bonferroni method. Again, the result showed the “Posterior w/prior” > “Posterior w/o prior” [t(15)= 9.98, p_correct_ < 10^-6^], “Posterior w/prior” > “Prior” [t(15) = -5.34, p_correct_ = 0.00025], and “Prior” > “Posterior w/o prior” [t(15) = -7.41, p < 10^-5^]. Those results demonstrate that the posterior sentences associated with prior sentences were more understandable than those without prior sentences. In addition, posterior sentences themselves were difficult to understand compared with prior sentences, but the associations with prior sentences made the posterior sentences more understandable than prior sentences alone. Therefore, our premise was satisfied.

Next, we explored the factors that influenced the understanding of posterior sentences associated with prior sentences. Focusing on such associated sentences, we investigated the contributions to the understanding of posterior sentences by “the understanding of prior sentences,” “the expected increment of prior understanding,” “the importance,” and “the subjective need” (see section “Prior Sentence Rating” in Materials and Methods). Using repeated measures of ANCOVA with reported degrees of understanding for the posterior sentences as the dependent variables, we observed a main effect of “the understanding of prior sentences” [F(1, 158) = 4.47, p = 0.036]. The other variables did not result in significant effects. These findings also demonstrate that prior knowledge augments the ability to acquire related information in the present experimental design.

### Contributions of the Prototypical Neural Representations for Sentence Comprehension

Our main concern in this study was the effect of prototypical neural representations (or preplay-like activity) observed in the rsfMRI prior to the posterior sentence presentation on the successive comprehension of the sentences. To this end, we applied representational similarity analysis (Kriegeskorte, 2008; Kriegeskorte and Kievit, 2013). To identify the neural representations for posterior sentences, we acquired stimulus-induced fMRI while the subjects read each posterior sentence and imagined its meaning. Some of the posterior sentences were associated with prior sentences, while others did not have any association. To measure the intensity of preplay-like activity, we calculated the correlations between the identified neural representations and the activity patterns in the *Day 2 pre* rsfMRI (light blue in **Figure 1A**). We defined the mean of the top 15 values of the Fisher-transformed correlations as the intensity of preplay-like activity for each posterior sentence (*N*_top_ = 15; see section “Representational Similarity Analysis”) (**Figure 1B**). We set the merged area of the hippocampus and entorhinal cortex as the ROI (see section “Representational Similarity Analysis”).

To test our main hypothesis and investigate the contributions of prior knowledge, we compared the intensities of preplay-like activity in the *Day 2 pre* rsfMRI and the degrees of understanding for the posterior sentences with and without the association of prior sentences (**Figure 2A** and see Supplementary Table S2 to survey data combinations used in each analysis). We found a weak but significant correlation between these two variables only when the associations with prior sentences were available (r_merged_ = 0.17, p_incorrect_ = 0.023, p_correct_ = 0.046 for “w/prior” and r_merged_ = 0.020, p_uncorrect_ = 0.42, p_correct_ = 0.85 for “w/o prior”), although direct comparison of the correlations resulted in no significant difference (p = 0.13) (but see section “Roles of the Entorhinal Cortex”). Next, we separated the posterior sentences into those with preplay-like activity intensity above-average or below-average (**Figure 2B**). We observed that preplay-like activity brought facilitating effects for understanding (a higher intensity of preplay-like activity resulted in a higher understanding) only if associations with prior sentences were available [t(15) = 2.26, p_uncorrect_ = 0.020, p_correct_ = 0.039 for “w/prior” and t(15) = 0.051, p_uncorrect_ = 0.48, p_correct_ = 0.96 for “w/o prior”]. Additionally, we executed the same analyses with N_top_ = 45 (see section “Representational Similarity Analysis”) and obtained qualitatively invariant but moderately strong results (Supplementary Figure S1). The result of the correlation analysis was as follows: r_merged_ = 0.18, p_incorrect_ = 0.017, p_correct_ = 0.035 for “w/prior” and r_merged_ = 0.0007, p_uncorrect_ = 0.50, p_correct_ = 0.99 for “w/o prior.” The result of the comparison between above-average and below-average preplay-like activity was as follows: t(15) = 2.72, p_uncorrect_ = 0.0079, p_correct_ = 0.016 for “w/prior” and t(15) = -0.14, p_uncorrect_ = 0.56, p_correct_ = 1 for “w/o prior.” These results support our main hypothesis, arguing that the prototypical neural representations have a facilitating effect on knowledge acquisition when prior knowledge exists. In contrast, we did not observe any effect in the cases without prior sentences.

To further investigate the influence of prior knowledge, we applied a similar analysis to preplay-like activity for prior sentences extracted from the *Day 1* rsfMRI (**Figures 3A,B**). In this setting, we did not provide any prior information about the sentences before their presentation. Consistently, we observed no correlation between the intensities of preplay-like activity and the degrees of understanding for prior sentences (r_merged_ = 0.020, p = 0.42). We also did not find a significant difference between the understanding of prior sentences with above-average preplay-like activity and the understanding of prior sentences with below-average preplay-like activity [t(15) = -0.58, *p* = 0.72].

Together, our results suggest that preplay-like activity contributes to knowledge acquisition only if prior knowledge is available.

### No Contribution of the Replay-Like Activity for Prior Sentences to Posterior Sentence Comprehension

The results described in the previous section support our main hypothesis that preplay-like activity facilitates knowledge acquisition. However, we must also consider the other hypothesis claiming that a replay-like activity for prior sentences results in the facilitation observed above. In this case, in the posterior sentence fMRI, the subjects might remember the associated prior sentences. Thus, the activity that we previously showed that contributed to the understanding of the posterior sentences might not be “preplay-like” activity for the posterior sentences but “replay-like” activity for the prior sentences. If this idea is correct, when using the authentic replay-like activity, we should be able to observe the same relationship as was seen in the previous section. In other words, there should be a positive contribution of the replay-like activity for prior sentences to the understanding of the associated posterior sentences.

To examine this possibility, we extracted replay-like activity data for prior sentences from the *Day 2 pre* rsfMRI and tested the effects on understanding of posterior sentences (**Figures 4A,B**). We observed no positive correlation between these factors (r_merged_ = -0.065, p = 0.77). Additionally, in the analysis comparing the posterior sentences with above- and below-average replay-like activity for prior sentences, we found no difference in the understanding [t(15) = 0.40, *p* = 0.35]. Therefore, we did not find evidence supporting the counter hypothesis. However, we observed a positive relationship between the replay-like activity for prior sentences and the understanding of prior sentences (**Figure 4C**). In the analysis comparing prior sentences with above- and below-average replay-like activity, the understanding of the former was higher than that of the latter [t(15) = 2.06, p = 0.029]. Taken together, there was no contribution of the replay-like activity for prior sentences to the comprehension of the posterior sentences, but there was a relationship with the understanding of prior sentences themselves.

### Prior Experience Arranges the Preformed Activity to Operate as the Prototypical Neural Representation

We investigated whether the acquisitions of prior sentences resulted in the *de novo* formation of organized patterns of neural activity for prototypical neural representations. If so, the preplay-like activity for the posterior sentences associated with prior sentences should be more strongly established than that of sentences with no association. This effect should be observed as an increased intensity of preplay-like activity in the former case compared to the latter. Using a one-sided test, we examined the prediction by comparing the mean intensity of preplay-like activity for posterior sentences associated with prior sentences to that of posterior sentences not associated with prior sentences (**Figure 5A**). We found no significant difference [t(15) = -0.21, p = 0.58]. Additionally, to further test the effect of prior sentence acquisition, we investigated the differences in preplay-like activity for the posterior sentences in the *Day 1* rsfMRI and those in the *Day 2 pre* rsfMRI (**Figure 5B**). We found only marginally significant increases if the posterior sentences associated with the prior sentences [t(15)=2.08, p_uncorrect_ = 0.028, p_correct_ = 0.056 for “w/prior”], and no effect if no association was available [t(15) = 1.71, p_uncorrect_=0.054, p_correct_ = 0.11 for “w/o prior”]. These results suggest that the acquisition of prior sentences does not strongly lead to *de novo* formation of organized neural activity for prototypical representations, but merely arranges the preformed, or pre-existing activity to contribute to assimilation of the upcoming posterior sentences.

### Stabilizations of the Neural Representations Through Experience

We next asked whether stabilization of representation through actual experience, which is observed in the rodent brain, occurred in our experiment. We compared the intensities of preplay-like and replay-like activities for the posterior sentences (**Figure 6**). We extracted the replay-like activity for the *Day 2 post* rsfMRI with the same method as that for the preplay-like activity. As expected, the intensity of replay-like activity was higher that of the preplay-like activity extracted from the *Day 2 pre* rsfMRI for posterior sentences both associated and not associated with prior sentences [t(15) = 4.51, p_uncorrect_ = 0.0002, p_correct_ = 0.0004 for “w/prior” and t(15) = 2.47, p_uncorrect_ = 0.013, p_correct_ = 0.026 for “w/o prior”]. Additionally, we executed two-way repeated ANOVA with the TIME (*Day 2 pre* and *Day 2 post* rsfMRI) and PRIOR factor (with and without associated prior sentences). Consistently, we observed a significant main effect of the TIME [F(1, 621) = 20.56, p < 10^-5^], suggesting that the replay-like activity in the *Day 2 post* rsfMRI was more stable than the preplay-like activity in the *Day 2 pre* rsfMRI. We did not observe any main effect of PRIOR [F(1, 621) = 0.006, p=0.94] or an interaction between TIME and PRIOR [F(1, 621)=1.25, p = 0.26]. These findings suggest the stabilization of neural representation through actual experience.

### Roles of the Entorhinal Cortex

Next, we further explored the brain areas contributing to the effects that we have observed thus far. We separately analyzed the hippocampus and the entorhinal cortex to determine which contributed to the facilitating effect of understanding of posterior sentences by the prototypical neural representation (**Figure 7**). We found positive correlations between preplay-like activity (in the *Day 2 pre* rsfMRI) and the understanding of posterior sentences most strongly in the entorhinal cortex. As with the previous observation, the correlation was present only if the posterior sentences were associated with prior sentences (entorhinal cortex: r_merged_ = 0.24, p_uncorrect_ = 0.0029, p_correct_ = 0.012 for “w/prior” and r_merged_ = -0.045, p_uncorrect_ = 0.67 for “w/o prior”; hippocampus: r_merged_ = 0.11, p_uncorrect_ = 0.098 for “w/prior” and r_merged_ = -0.048, p_uncorrect_ = 0.68 for “w/o prior”). Moreover, direct comparison of the correlations between conditions with and without prior sentences resulted in a significant difference only in the entorhinal cortex (entorhinal cortex: p_uncorrect_ = 0.017, p_correct_ = 0.034; hippocampus: p_uncorrect_ = 0.12, p_correct_ = 0.23). Therefore, the entorhinal cortex, but not the hippocampus, plays a role in the effect observed above.

### Whole-Brain Exploration

To probe brain regions that are involved with the facilitation of understanding of the sentences by the preplay-like activity beyond the hippocampus and entorhinal cortex, we conducted the exploratory analysis of the whole brain. Using whole-brain GLM analysis, we investigated the correlation between the preplay-like activity of the posterior sentences in the *Day 2 pre* rsfMRI and the understanding of the posterior sentences for each voxel in the brain. As a result, we did not observed any area showing positive correlation between them regardless of associations with prior sentences. However, we found the areas in which the correlations for the posterior sentences associated with prior sentences are significantly higher compared with those without associated prior sentences (**Figure 8** and **Table 1**). These included the medial prefrontal cortex (mPFC) centered on the paracingulate gyrus, the superior frontal gyrus, and the juxtapositional lobule cortex.

**Table 1:** Significant clusters of the exploratory analysis of the whole brain.

	MNI coordinates				
Region	*x*	*y*	*z*	Number of voxels	*p*-value (corrected)
SFG	0	26	59	141	0.003
PCG	3	44	29	23	0.006
JLC	6	5	56	9	0.039

*SFG, superior frontal gyrus; PCG, paracingulate gyrus; JLC, juxtapositional lobule cortex.*

## Discussion

In the present study, we aimed to shed light on the neural mechanisms underlying selectivity in knowledge acquisition. Many previous psychological and neuroscience studies have suggested a rational in the acquisition of knowledge, in which we actively select information from the environment to upgrade our current knowledge.

We predominantly focused on the involvement of prototypical neural representations, or preplay-like activity, in knowledge acquisition. We used sentence comprehension as an instance of knowledge acquisition. We completed a 2-day experiment in which subjects were tasked with understanding prior and posterior sentences. In this situation, the prior sentences that were learned by the subjects were considered to be prior knowledge that contributed to the upcoming experience, which was acquisition of posterior sentences (upcoming knowledge acquisition). To investigate the relationship between acquisition of the posterior sentences and the neural activity in during the rest period before acquisition, we obtained a resting-state as well as a stimulus-induced fMRI. First, we showed that each posterior sentence was difficult to understand alone, but that association with prior sentence increased understanding of it. This demonstrates an effect of prior knowledge on facilitating sentence comprehension during our experimental procedure. Next, we investigated an effect of prototypical neural representations to the comprehension of posterior sentences with or without associations to prior sentences. Our results suggest that prototypical neural representations (preplay-like activity) leads to selectivity in knowledge acquisition by facilitating the acquisition of items according to the intensity of their respective prototypical neural representations only if the posterior sentences were associated with the prior sentences. Even though the subjects did not know the concrete posterior sentences when prototypical neural representations appeared, the prototypical neural representations represented these sentences. Therefore, prototypical neural representations are proactive and can serve as a substitute for an as-yet-unknown experience. This means that prototypical neural representation is useful for predicting the future and decreasing uncertainty. Moreover, our results suggest that the brain spontaneously pre-selects what should be learned. An existence of prior knowledge is vital for this mechanism to operate since the facilitating effect by the prototypical neural representation was observed only if prior knowledge was available. Most likely, the brain represents the pre-selected item as a prototypical neural representation based on prior knowledge. Probably, this is selectively acquired through upcoming actual experience.

In the present study, we did not find evidence to suggest that prior knowledge resulted in newly formed organized patterns of neural activity. Rather, our observations suggest that prior knowledge influences the arrangement of preformed patterns and enable them to become a prototypical neural representation as the basis for an upcoming knowledge acquisition. Theoretical studies suggest that sufficiently complex neural networks include rich patterns of activity, which enables the networks to realize arbitrary computation (Maass et al., 2002; Jaeger and Haas, 2004; Sussillo and Abbott, 2009; Hoerzer et al., 2014; Rajan et al., 2016; Thalmeier et al., 2016). Therefore, it is reasonable to postulate that the brain has intrinsically sufficient neural patterns that are arranged according to prior knowledge for future neural representations.

We also showed that prototypical neural representation is stabilized and becomes more established through an actual experience. This result is similar to the phenomenon observed in the rodent brain (Grosmark and Buzsaki, 2016). However, this may be simply caused by the temporal proximity of the measurement of the stimulus-induced fMRI for posterior sentences and the *Day 2 post* rsfMRI for replay-like activity. Further studies are required to clarify which interpretation is more feasible.

We found the entorhinal cortex, rather than the hippocampus, to be the primary contributor of the effects that we have mentioned. In rodent studies, the involvement of the entorhinal cortex has been investigated with respect to replay, but not preplay. Thus, our study provides novel evidence of the areas involved in preplay activity. The time elapsed since the initial learning experience may be a possible factor influencing the involvement of specific areas of the brain, as the progress of learning and consolidation may shift the processing center from the hippocampus to the entorhinal cortex (Takehara-Nishiuchi et al., 2012).

In addition, the exploratory analysis of the whole brain suggested that several brain areas contribute the facilitating effect of the prototypical neural representation on understanding of the posterior sentences when prior sentences are available. Especially, the involvement of the mPFC is noteworthy since several previous studies suggest that mPFC plays a role in memory recall and consolidation when congruent prior knowledge exists (van Kesteren et al., 2010b, 2013, 2014; Tse et al., 2011). Therefore, this result may suggest that similar neural mechanism underlies the facilitation of the understanding of the sentences observed in the present study.

However, it is worthwhile to note that our findings in the human brain do not parallel the findings reported in the original preplay paper (Dragoi and Tonegawa, 2011). First, in the present study, we showed that the prototypical neural representation contributes to upcoming knowledge acquisition, but we did not examine how frequently such prototypical neural representations exist. On the other hand, in the original preplay paper, the authors suggested that preplay activity exists significantly more than random patterns. Second, we demonstrated the role of prior knowledge on prototypical neural representation, while the original preplay paper focused on naïve animals assumed to have no prior knowledge. Therefore, we do not argue that the prototypical neural representation observed in the present study accurately corresponds to preplay activity in the rodent brain. Further studies are required to clarify the relationship between prototypical neural representations that we investigated and preplay activity in the rodent brain.

### Possible Mechanisms

**FIGURE 9:**
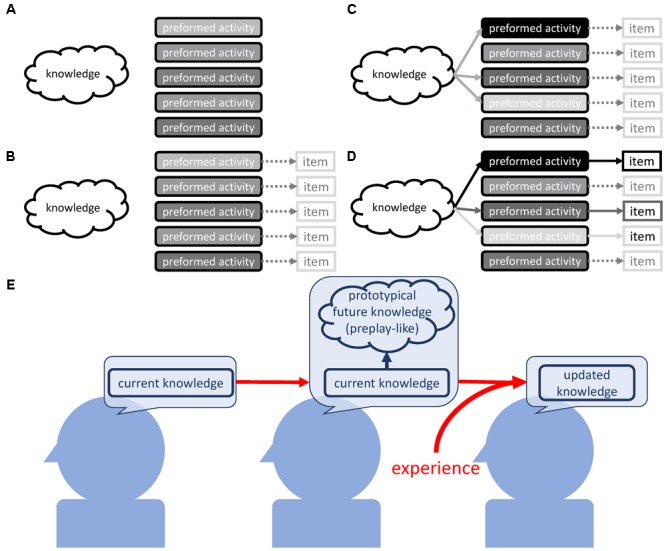
Possible mechanism. **(A)** Preformed organized neural activity as part of the self-organized nature of the neural circuit. **(B)** Rough contingent associations between preformed neural activity and external items. **(C)** Prior knowledge modulates the intensities of preformed neural activity, which is the arrangement process for the activities as prototypical neural representations. **(D)** The encounter of the preformed activity (that is now the prototypical representation) and the concrete external item. This induces resonance between prior knowledge and the preformed activity, and leads to the assimilation of the item into the current knowledge. This is reflected in the strengthening of the link between knowledge and neural activity pointing to the particular item. **(E)** Current knowledge arranges the prototypical neural representation as a designated position for future knowledge. The experience that fits this prototypical neural representation is easily assimilated.

Our findings suggest the existence of a two-step mechanism that results in selectivity of knowledge acquisition through prototypical neural representations that are arranged based on prior knowledge (**Figure 9**). We will first brief the possible mechanism and then explain it in depth.

As the first step, we assume the existence of preformed organized brain activities that are rich enough to cover the items that we possibly assimilate into our knowledge (**Figure 9A**). These may become prototypical neural representations at a later stage. At the second step, the preformed brain activity is arranged to operate as prototypical neural representations with the intensity determining the understanding or assimilation of the items they will later represent. This involves sub steps; at the first sub step, the preformed activity contingently has loose coupling with an as-yet-unknown external item (**Figure 9B**). Then, prior knowledge modulates the preformed activities and strengthens the ones that correspond to the items to be learned (**Figure 9C**). Now, the preformed activity operates as prototypical neural representations and contributes to the selectivity of knowledge acquisition. Finally, actual encounters of the prototypical neural activity and concrete items that are strongly represented by it result in the strong assimilation of the item into knowledge (**Figure 9D**).

Next, we will give an in-depth explanation of each of these steps. For **Figure 9A**, the necessity of the preformed brain activity is based on the results suggesting that experience does not result in new formation of the pattern that operates as preplay-like activity. Rather, experience influences the arrangement of the preformed pattern of neuronal activity (**Figure 5**). In order to cover the multitude of possibilities that we assimilate into our knowledge on the basis of prototypical neural representations, the preformed activities should be highly diverse. We propose that two non-exclusive sources, non-random external stimuli and internally generated neural dynamics, could lead to such a development of pattern activity. The former is evident from the close comparisons between spontaneous brain activity and activity evoked with naturalistic stimuli. Indeed, in the ferret visual cortex, the statistics for spontaneous activity become similar to the ones for activity evoked by naturalistic stimuli (Berkes et al., 2011). This means that neural plasticity driven by the non-random naturalistic stimuli endows the brain with organized activity, and does so with such high diversity that it allows for coverage of the diversity in natural stimuli. Additionally, in the human visual cortex, one can also find such similarities (Wilf et al., 2017).

For the latter source, it is possible for the brain to form organized activity through activity-dependent plasticity that is recursively driven by internally generated neural dynamics (Rubinov et al., 2009, 2011; Miner and Triesch, 2016). One intriguing theoretical study showed that the recurrent neural networks with Hebbian synaptic plasticity and homeostatic intrinsic plasticity automatically grow into a dynamic regime called the “edge of chaos” under biologically plausible conditions (Naudé et al., 2013). The networks that make up the “edge of chaos” are known to possess the preferred characteristics for information processing (Bertschinger and Natschläger, 2004; Legenstein and Maass, 2007; Büsing et al., 2010; Boedecker et al., 2012). A high separation property seems to be especially beneficial for this step, as it reflects the ability to represent the effective diversity of the possible states.

Next, the preformed activity must be arranged to operate as prototypical neural representation. We observed that assimilation was influenced by the intensity of preplay-like activity, only if prior knowledge was available (**Figures 2A,B**). Thus, the arrangement occurs during and/or after the construction of prior knowledge.

Here, the tripartite relationship between prior knowledge, preformed activity, and an external item must be considered (**Figure 9B**). The preformed activity roughly or imaginarily points to the external items, as these items are not yet concrete. At least at this stage, prior knowledge is not able to modulate the correspondence between preformed activities and external items, as the physiological substance of the correspondence is simply the activation of neural patterns by the specific range of inputs (**Figure 9B**).

One may consider that we better assimilate items that are incidentally pointed out with intense preformed activity. However, the observation in the case of no prior knowledge (**Figures 2, 3**) disproves this idea. The strength of the connection between prior knowledge and a preformed activity seems to determine the resulting amount of the assimilation of the item. We simply assume that coactivation of prior knowledge and the preformed activity leads to a strong assimilation of such an item pointed out with the activity, like Hebbian plasticity (Hebb, 1949; Bliss and Collingridge, 1993). Low activation of prior knowledge explains the weak assimilation of the items with the unavailability of prior knowledge, regardless of the intensity of preformed activities.

Next, we can consider the two scenarios in which prior knowledge is available. In the first scenario, the items incidentally pointed out by the intense preformed activity are strongly assimilated. If true, the strength of assimilation would be ruled by chance. However, we observed that a strong understanding of prior sentence set off the strong understanding of associated posterior sentence, and. therefore, we should reject the first scenario. In the second scenario, prior knowledge modulates preformed activities and strengthens the ones corresponding to items to be learned (**Figure 9C**). In this scenario, the items pointed out with the preformed activities strengthened by prior knowledge are strongly assimilated (**Figure 9D**). This scenario successfully explains the positive tripartite relationship between prior knowledge, preformed activity, and assimilation of an external item. Thus, through modulation of the preformed activity, prior knowledge indirectly determines the item that we should learn from the environment.

### Implications for Cognitive Functions

In the present study, we showed the possible neuronal mechanism underlying selectivity in knowledge acquisition. If the brain actively selects the information and updates the knowledge according to the latent policy, knowledge acquisition is directional. Naturally, this introduces the view where we regard knowledge acquisition as the optimization process governed by objective functions. However, to reveal the computational principles in knowledge acquisition, we must know the objective functions.

Our results indicate the presence of a pathway between current and updated knowledge via generation of prototypical neural representations. Therefore, further investigation of this pathway may contribute to the identification of objective functions. In the present study, we could not find any neuronal identifier that discriminates the prototypical neural representations observed when prior knowledge is available from the false ones observed when prior knowledge is not available. As a next step, it is important to find the identifier.

In our experiment, prior sentences were difficult to understand alone and required associable posterior sentences to increase their understanding. This could mean that the prior sentences had semantic deficits. Therefore, prototypical neural representations may identify the deficit to be compensated. From this perspective, the items pointed out with self-generated prototypical neural representations are considered to be deficits in knowledge. What is recognized as a “deficit” by our brain is a vital factor that determines the direction in which the brain will lead us. We may gain insight into this mechanism by clarifying the generative process of prototypical neural representations as the icons for the deficits.

It is important to review our results in relation to “schema.” A schema is structured information or knowledge for a cluster of events constructed in our minds, and is a central component of neuroscience for learning and memory (Ghosh and Gilboa, 2014; Gilboa and Marlatte, 2017). In an early study, “slots,” or variables to be filled, were defined as the key concept featured in a schema (Minsky, 1975; Schank and Abelson, 1977; Rumelhart et al., 1986). There is a similarity between slots and prototypical neural representations observed in the present study. Since slots are preset positions in the current knowledge reserved for upcoming information, they enable the brain to process information in proactive, imaginary, or counterfactual manners. This may increase the brain’s readiness for future events. A recent theoretical study suggested that preplay activity enabled the neural circuit to do so-called “one-shot” learning (Haga and Fukai, 2017). Additionally, using prototypical representations instead of upcoming concrete information may enable us to think in advance.

The present study presents a view where we regard knowledge acquisition as a so-called autopoiesis. Autopoiesis is the process where a system autonomously and continuously regenerates itself (Maturana and Varela, 1980, 1987). From our observations, together with the previous research on knowledge acquisition, current knowledge generates prototypical future knowledge that probably satisfies some needs (**Figure 9E**). More precisely, current knowledge generates prototypical information as preplay-like activity that is the rough component of future knowledge. It serves as a pointer to point to what one should learn, and is the position reserved in advance for new information. The actual acquisition of such information actively reorganizes the existing knowledge. Further in-depth investigation of such an autopoietic nature will reveal how and why our knowledge is superior and defective, and will lead the development of artificial intelligence that can acquire knowledge autonomously.

### Limitations

In the present study, we used sentence comprehension as an instance of knowledge acquisition. The sentences that were provided to subjects were more or less philosophical. We postulate that the objective correctness in the comprehension of such sentences is decided not only from internal cognitive ability of knowledge acquisition, but also from external social norms and/or scientific facts that are out of the scope of this study. Therefore, we adopted self-ratings of understanding as the index of knowledge acquisition. As a result, the index relied on subjectivity of the subjects. Although we acknowledge that self-ratings of understanding probably correlate closely to the actual degrees of knowledge acquisition, we could adopt a more objective index. For instance, a follow-up memory test after 1 week may be a better method. Investigation using more objective indexes remains a task for future studies.

Relating to this issue, it is worthwhile to discuss the validity of use of sentence comprehension as an instance of knowledge acquisition. In the human, comprehension of sentences containing new information through reading or listening is a typical and natural way for knowledge acquisition. The posterior sentences used in this study were new for the subjects even if some parts were associated with the prior sentences. In other words, the subjects acquired new knowledge through comprehending the sentences. Therefore, the comprehension is inevitably considered to indicate knowledge acquisition. Additionally, previous researches suggest close relationship between language comprehension and knowledge acquisition (Maguire et al., 1999). Moreover, several studies suggest the involvement of hippocampal region into the language comprehension task, which means that language comprehension needs to form new knowledge or synaptic connections especially if the sentences are more or less new (MacKay et al., 1998; Awad et al., 2007; Duff and Brown-Schmidt, 2012). In our experimental setting, the posterior sentences were highly novel for the subjects. Therefore, our protocol probably led the subjects to make new knowledge by involving the hippocampus and the entorhinal cortex. Thus, our task was validate in the sense that it included knowledge acquisition as a main constituent.

Since our main concern of this study was relationship between preplay-like activity and knowledge acquisition, we mainly focused on the hippocampus and the entorhinal cortex. On the other hand, we had little concern for language areas. However, as our task was linguistic, it is expected that these areas play a role in the task. To investigate the involvement of these areas clearly, we first need to identify these areas in individual brain because of large anatomical ambiguities and intersubject variability of the borders (Juch et al., 2005). To overcome those difficulties, an experimental protocol including localization scans for language areas consisting of well-established language tasks are required. We consider such an experiment as a future issue.

Although we instructed subjects to think of nothing in particular before each measurement of rsfMRI, we could not eliminate the possibility that subjects recalled the learned sentences. Therefore, we may need to discount our findings about “replay”-like activity. However, our main concern, “preplay”-like activity, is not affected by this because the subjects did not know the sentences prototypically represented by the activity when we acquired the rsfMRI. Additionally, we showed no effect of replay-like activity for prior sentences on posterior sentence comprehension. Therefore, this issue does not diminish our main conclusion.

Finally, we should note the strength of our conclusion. Through the present study, our findings did not show exceptionally strong statistical significance, which could be partially caused by the complicated design of the task and a relatively small sample size (Button et al., 2013). We observed moderately strong results from the analysis with *N*_top_ = 45, and the analysis focused on the entorhinal cortex. However, we should discount these results because of weak but existing circularity (Kriegeskorte et al., 2009). The findings reported in the present study are highly novel. On the other hand, we do not have enough collateral evidence peripherally. Given these issues and the controversy about preplay activity mentioned in the introduction, further studies are required to augment the conclusion.

## Author Contributions

HK performed the experiments and data analysis. All authors contributed to the design of the study and writing of the manuscript.

## Conflict of Interest Statement

The authors declare that the research was conducted in the absence of any commercial or financial relationships that could be construed as a potential conflict of interest.

## Footnotes

1 https://wxpython.org/

2 https://surfer.nmr.mgh.harvard.edu/

3 http://www.fmrib.ox.ac.uk/fsl/

4 https://jp.mathworks.com/matlabcentral/fileexchange/8797-tools-for-nifti-and-analyze-image

5 https://www.scipy.org/

6 http://www.statsmodels.org/

7 https://www.r-project.org/
