# Supplementary material for: A Knowledge-Based Arrangement of Prototypical Neural Representation Prior to Experience Contributes to Selectivity in Upcoming Knowledge Acquisition
Source: Front Hum Neurosci. 2018 Mar 21;12:111. doi: 10.3389/fnhum.2018.00111 (PMC5890192; doi:10.3389/fnhum.2018.00111)
Supplement: Supplementary file 3 [file Data_Sheet_1.docx]

Supplementary Material

A Knowledge-based Arrangement of Prototypical Neural Representation Prior to Experience Contributes to the Selectivity in Upcoming Knowledge Acquisition

Hiroki Kurashige^1,2*^, Yuichi Yamashita^2^, Takashi Hanakawa^3^, Manabu Honda^2^

*** Correspondence:** Hiroki Kurashige: h.kura00@gmail.com


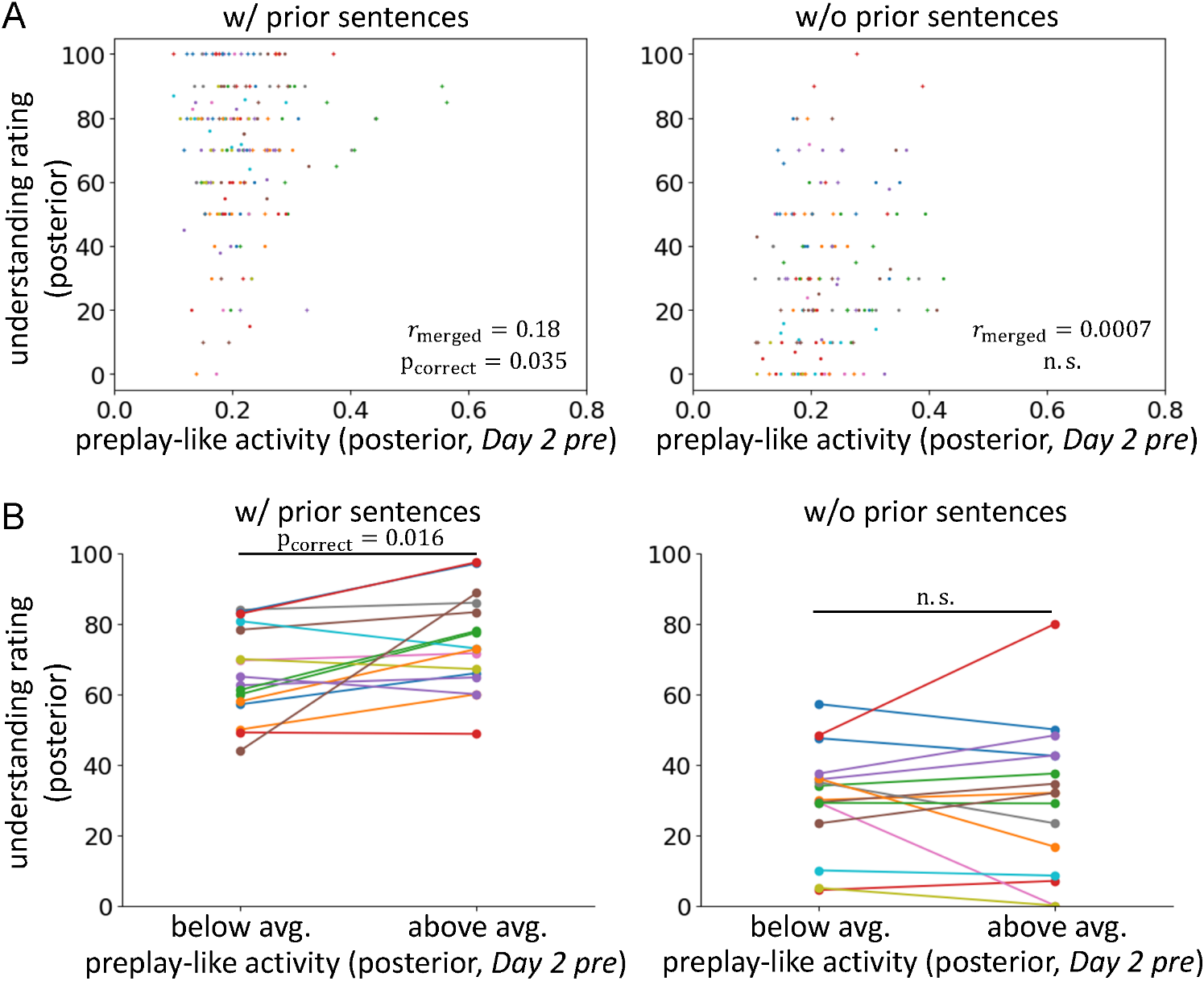


**Supplementary Figure 1.** Same to Figure 1 but $N_{\mathrm{top}}=45$.
